# Supplementary material for: iNP_ESM: Neuropeptide Identification Based on Evolutionary Scale Modeling and Unified Representation Embedding Features
Source: Int J Mol Sci. 2024 Jun 27;25(13):7049. doi: 10.3390/ijms25137049 (PMC11240975; doi:10.3390/ijms25137049)
Supplement: Supplementary file 1 [file ijms-25-07049-s001.zip › ijms-3051030-supplementary.pdf]

International Journal of *Molecular Sciences*

MDPI

Table S1 Comparison of independent test accuracy for six encoding methods across seven machine learning algorithms.

Metrics	Independent test_ACC						
**Features/Algorithms**	**GNB**	**KNN**	**LDA**	**LGBM**	**LR**	**RF**	**SVM**
UniRep	0.736	**0.885**	0.889	0.907	0.894	**0.896**	0.904
ESM	**0.777** [1]	0.882	**0.898**	**0.914**	**0.902**	0.892	**0.918**
SSA	0.633	0.825	0.806	0.871	0.803	0.855	0.875
LM	0.766	0.833	0.868	0.897	0.865	0.880	0.898
BiLSTM	0.744	0.849	0.841	0.903	0.870	0.883	0.906
TAPE_BERT	0.760	0.851	0.880	0.877	0.864	0.865	0.896

[1] The best value of each column is underline and in bold.

Table S2 Comparison of 10-fold cross-validation metrics for LGBM and SVM-based models using fused feature and single feature (parameter optimized).

**Algorithms**	**LGBM**							**SVM**						
**Features/Metrics**	**ACC**	**MCC**	**Sn**	**Sp**	**Pre**	**F1**	**AUC**	**ACC**	**MCC**	**Sn**	**Sp**	**Pre**	**F1**	**AUC**
UniRep (optimized)[1]	0.927	0.855	0.947	0.907	0.911	0.929	0.980	0.932	0.864	0.937	**0.927**	**0.928**	0.932	0.980
ESM (optimized)[2]	0.920	0.840	0.936	0.903	0.907	0.921	0.976	0.928	0.857	0.932	0.924	0.925	0.929	0.978
UniRep+ESM_F3180[3]	**0.932** [4]	**0.866**	**0.950**	**0.915**	**0.918**	**0.934**	**0.982**	**0.933**	**0.867**	**0.942**	0.925	0.927	**0.934**	**0.981**

[1] LGBM parameters: {'learning_rate': 0.05, 'max_depth': 12, 'n_estimators': 800}SVM parameters: {'C': 17.433288221999874, 'gamma': 0.0012689610031679222, 'kernel': 'rbf'}[2] LGBM parameters: {'num_trees': 500, 'learning_rate': 0.28}SVM parameters: {'C': 14.677992676220706, 'gamma': 0.001}[3] LGBM parameters: {'num_trees':1300, 'learning_rate': 0.28}SVM parameters: {'C': 1.9306977288832496, 'gamma': 'scale'}

[4] The best value of each column is underline and in bold.

Table S3 Comparison of independent test metrics for LGBM and SVM-based models using fused feature and single feature (parameter optimized).

**Algorithms**	**LGBM**							**SVM**						
**Features/Metrics**	**ACC**	**MCC**	**Sn**	**Sp**	**Pre**	**F1**	**AUC**	**ACC**	**MCC**	**Sn**	**Sp**	**Pre**	**F1**	**AUC**
UniRep (optimized)	0.913	0.827	**0.930**	0.896	0.900	0.915	0.973	0.920	0.840	0.917	0.923	0.923	0.920	0.971
ESM (optimized)	0.914	0.829	0.926	0.903	0.905	0.915	0.971	0.919	0.838	0.912	**0.926**	0.925	0.918	0.971
UniRep+ESM_F3180	**0.926** [1]	**0.851**	**0.930**	**0.921**	**0.922**	**0.926**	**0.976**	**0.928**	**0.856**	**0.930**	**0.926**	**0.926**	**0.928**	**0.977**

[1] The best value of each column is underline and in bold.

Table S4 Comparison of 10-fold cross-validation metrics between iNP_ESM and NeuroPred-PLM.

**Methods**	**ACC**	**MCC**	**Sn**	**Sp**	**Pre**	**F1**	**AUC**
iNP_ESM_F3180	0.933	0.867	**0.942**	0.925	0.927	**0.934**	**0.981**
iNP_ESM_F120	**0.937** [1]	**0.873**	0.940	**0.933**	**0.980**	**0.934**	0.937
NeuroPred-PLM	0.927	0.854	0.928	0.926	0.926	0.927	-

[1] The best value of each column is underline and in bold.
